# The Role of Long Non-Coding RNA in the Pathogenesis of Psoriasis

**DOI:** 10.3390/ncrna11010007

**Published:** 2025-01-17

**Authors:** Kajetan Kiełbowski, Anna Jędrasiak, Estera Bakinowska, Andrzej Pawlik

**Affiliations:** Department of Physiology, Pomeranian Medical University, 70-111 Szczecin, Poland; annajedrasiak99@gmail.com (A.J.); esterabakinowska@gmail.com (E.B.)

**Keywords:** psoriasis, long non-coding RNA, keratinocyte proliferation, inflammation

## Abstract

Psoriasis is a chronic immune-mediated disease with complex pathogenesis. The altered proliferation and differentiation of keratinocytes, together with the activity of dendritic cells and T cells, are crucial drivers of psoriasis progression. Long non-coding RNAs (lncRNAs) are composed of over 200 nucleotides and exert a large variety of functions, including the regulation of gene expression. Under pathological conditions, the expression of lncRNAs is frequently dysregulated. Recent studies demonstrated that lncRNAs significantly affect major cellular processes, and their aberrant expression is likely involved in the pathogenesis of various disorders. In this review, we will discuss the role of lncRNAs in the pathophysiology of psoriasis. We will summarize recent studies that investigated the relationships between lncRNAs and keratinocyte proliferation and pro-inflammatory responses.

## 1. Introduction

Psoriasis is a chronic and relapsing inflammatory dermatosis. Recent epidemiological studies show that the prevalence of psoriasis varies between 0.14% and 6.60%, with an average prevalence of approximately 2% in the global population [1]. This condition affects approximately 125 million people worldwide [2], and varies in frequency depending on geographical location and ethnic demographics, with an increased prevalence in Western Europe and decreased prevalence in some regions of Asia [3]. In the United States, psoriasis affects approximately 7.5 million people [4], and the financial impact of its treatment is significant, mainly due to systemic complications. The annual economic impact of psoriasis in the United States is estimated to be approximately USD 35.2 billion [5]. The disease often leads to limitations in work capacity, reducing productivity and increasing sickness absence, leading to economic losses. In addition, the treatment of psoriasis, especially with modern biologic drugs, is expensive, placing a heavy burden on the healthcare system [6].

**Figure 1:**
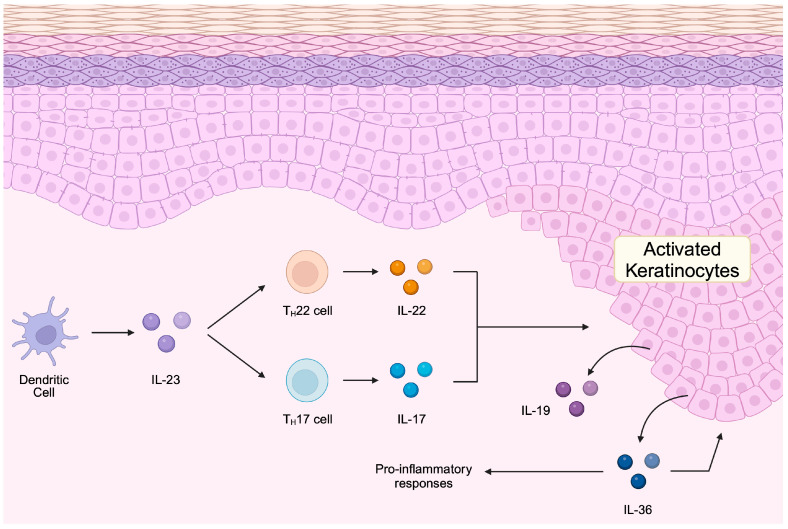
A simplified model of immune cells implicated in the pathogenesis of psoriasis. Dendritic cells secrete IL-23, which induces the differentiation of Th22 and Th17 cells. Cytokines secreted by differentiated T cells induce keratinocyte activation and drive the development of psoriatic lesions. Created in BioRender. Kiełbowski, K. https://BioRender.com/p62u479.

The pathophysiological mechanisms underlying psoriasis involve a complex interplay of genetic and immunological components. Thousands of transcripts are differently expressed between the lesional, non-lesional, and healthy skin [7]. Psoriasis is classified as an inflammatory disease in which the dysregulation of the immune response plays a key role. Studies suggest that psoriasis is characterized by the activation of dendritic cells and T lymphocytes [8], culminating in the synthesis of pro-inflammatory cytokines such as interleukin (IL)-17, IL-23, and TNF-α [9]. These cytokines induce an excessive increase in keratinocyte proliferation, insufficient keratinization, and a pro-inflammatory environment in the dermis, which ultimately result in the characteristic pathological manifestations, including erythematous and scaly lesions [10,11,12,13]. Stimulated keratinocytes release immunoregulatory mediators such as IL-19 and IL-36 [14,15] (Figure 1). Furthermore, this condition is associated with an elevated risk of various severe comorbidities, particularly psoriatic arthritis and cardiovascular disorders [16]. The prevalence of cardiovascular disease among individuals with psoriasis is 25% higher than in the general population [17].

Non-coding RNA (ncRNA) is a broad family of RNA molecules deeply involved in regulating gene expression and consequently major cellular behaviors. According to the recently published consensus statement by Mattick et al. [18], ncRNA are structurally divided into three subgroups. The first one involves small RNAs (<50 nucleotides) and the second includes transcripts of RNA polymerase III (Pol III), Pol V transcripts in plants and small Pol II transcripts (50–500 nucleotides). The third group is composed of lncRNAs. ncRNA molecules can be considered lncRNA when they are at least 500 nucleotides long. Taking into account protein-coding genes, lncRNAs can be classified into three principal categories: antisense transcripts, which are transcribed from sequences that are complementary to protein-coding genes; intronic RNAs, which are derived from introns; and long intergenic RNAs, which are located between genes [18]. Increasing evidence suggests that lncRNAs play a pivotal role in a multitude of biological processes. Dysregulation of these molecules is frequently linked to a range of pathological conditions, including psoriasis. They have the ability to induce changes in gene expression in a locus-specific manner through recruitment of chromatin modifying factors [1,19]. The importance of DNA methylation, histone modifications and the activity of members of the ncRNA family is getting an increased attention in studies investigating pathogenesis of psoriasis. The influence of genetics and epigenetics was elegantly summarized in a 2020 review paper written by Nedoszytko et al. [20].

Under pathological conditions, the expression of numerous lncRNA molecules is dysregulated. Some lncRNAs, including metastasis-associated lung adenocarcinoma transcript-1 (MALAT-1) and psoriasis-susceptibility-related RNA gene induced by stress (PRINS), have been demonstrated to influence keratinocyte proliferation and inflammatory responses, thereby engaging in regulatory mechanisms of psoriasis pathogenesis [21,22]. lncRNAs regulate pivotal signaling pathways linked to immune responses and inflammatory processes. In the context of psoriasis, they are becoming increasingly significant in the investigation of the etiology of this disease and represent a promising area for the development of novel therapeutic approaches. The objective of this review is to present the role of lncRNAs in the pathophysiology of psoriasis and to discuss their potential as diagnostic and therapeutic biomarkers.

## 2. Long Non-Coding RNAs in the Pathogenesis of Psoriasis

Over the years, numerous studies have demonstrated the dysregulation of lncRNA molecules in patients with inflammatory and autoimmune diseases [23]. In psoriasis, one of the recent analyses demonstrated over 1200 differentially expressed lncRNAs between lesional psoriatic and normal skin [24]. Many aberrantly expressed lncRNAs remain uncharacterized [25]. These findings suggest the involvement of lncRNAs in the pathophysiology of diseases or compensatory mechanisms induced by an organism. lncRNAs are involved in the regulation of gene expression, often affecting the behavior of keratinocytes and immune cells.

Complex interactions between keratinocytes and immune cells and cytokines induce the proliferation of keratinocytes, a hallmark feature of psoriasis [26]. Researchers demonstrated that numerous stimulants enhance keratinocyte proliferation, including high levels of double-stranded DNA fragments [27], adipokines [28], mechanical stretch [29], and inflammatory molecules [30], among many others. Accordingly, targeting keratinocyte proliferation is considered beneficial in the treatment of psoriasis lesions [31,32].

Immune cells secrete cytokines that significantly affect the behavior of keratinocytes, thus driving the progression of psoriasis. Dendritic cells, T cells, activated keratinocytes, nuclear factor kappa B (NF-κB) transcription factor, IL-23, IL-21, IL-22, and IL-17 are some of the important immune-related elements involved in the pathophysiology of psoriasis. Frequently, the stimulation of immune responses is also associated with the enhancement of keratinocyte proliferation.

In studies investigating the pathogenesis of psoriasis, the expression of lncRNA molecules has been demonstrated to undergo significant alterations in both blood and skin biopsies obtained from patients. The utilization of bioinformatics methodologies has facilitated the identification of pivotal lncRNAs that may possess diagnostic and therapeutic implications, including the capacity to predict treatment response [33].

To begin with, studies demonstrated the altered expression of lncRNA PRINS. Specifically, the molecule was found to be downregulated and upregulated in leukocytes and skin from psoriasis patients, respectively [34,35]. Furthermore, PRINS expression is observed to decline during successful psoriasis treatment [36]. These findings suggest that the molecule could be involved in the pathogenesis of psoriasis. Early studies demonstrated an important role of PRINS in keratinocyte functionality. By regulating the expression of G1P3, it mediates skin cell apoptosis [36,37]. Additionally, PRINS is associated with cellular stress [36]. It binds to nucleophosmin (NPM), which is involved in responses to environmental stress and mRNA processing. Moreover, its expression in keratinocytes fluctuates as these cells differentiate [37]. NPM is upregulated in psoriasis. It is released from stimulated keratinocytes and it induces pro-inflammatory responses. Circulating NPM showed a positive correlation with the PASI score [38].

lncRNA activated by DNA damage (NORAD) has been recently implicated in the process of cellular proliferation. lncRNA molecules are frequently widely investigated in an oncological context. NORAD enhances the proliferation of several malignancies, including hepatocellular carcinoma, lymphoma, and pancreatic cancer [39,40,41]. However, NORAD was found to mediate proliferation in non-cancerous tissues as well. Through binding to miR-150-5p, NORAD promotes the proliferation of cardiomyocytes [42]. The activity of NORAD was also tested in psoriasis in in vitro and in vivo experiments. Specifically, keratinocytes stimulated with IL-22/LPS showed the upregulation of NORAD. Similarly, the expression of lncRNA was also increased in an IMQ-treated mouse model. The upregulation of NORAD was not a compensatory mechanism but a pathophysiological mechanism. The introduction of si-NORAD reduced keratinocyte proliferation and cell viability. Mechanistically, it was suggested that NORAD interacts with miR-26a and thus regulates the expression of cell division cycle 6 (CDC6) [43]. Importantly, another study demonstrated that the treatment of a psoriasis mouse model with miR-26a-5p reduced skin thickness, together with scaling and erythema scores. CDC6 is an important mediator of DNA replication [44], which explains the involvement of the NORAD/miR-26a/CDC6 axis on keratinocyte proliferation. CDC6 is involved in the mechanisms mediated by cyclin-dependent kinases (CDKs). We discussed the potential role of CDKs in psoriasis in our previous paper [45]. The expression of CDC6 is regulated by IL-22, one of the important cytokines implicated in the pathogenesis of psoriasis [46]. To the best of our knowledge, it is unknown whether IL-22 directly downregulates miR-26a to induce the effect on CDC6. Nevertheless, the expression of miR-26a is reduced in IL-22-secreting T cells [47]. IL-22 can potentially act in various mechanisms by upregulating and downregulating NORAD and miR-26a, respectively. IL-22 also induces the expression of the lncRNA MALAT1, which acts through miR-330-5p and SMAD7 to induce keratinocyte proliferation [21]. MALAT-1 is a long-known lncRNA whose role has been extensively examined in the context of lung cancer [48]. However, its presence and potential function has been established in inflammatory conditions as well. The expression of this molecule is elevated in lesional skin in psoriasis patients in comparison to non-lesional skin [49,50]. Harboring particular genetic variants of the MALAT1 gene is also associated with altered risk of the disease [51]. Hypothetically, immunoregulatory properties of MALAT1 could be implicated in the pathogenesis of the disease. Other studies showed that this lncRNA can alter T cell differentiation, with evidence supporting its inhibiting [52] and enhancing [53] role in the process of Th17 differentiation. Thus, the precise role of MALAT1 could be context-dependent. To the best of our knowledge, the immunoregulatory properties of MALAT1 were not examined in the context of psoriasis.

**Figure 2:**
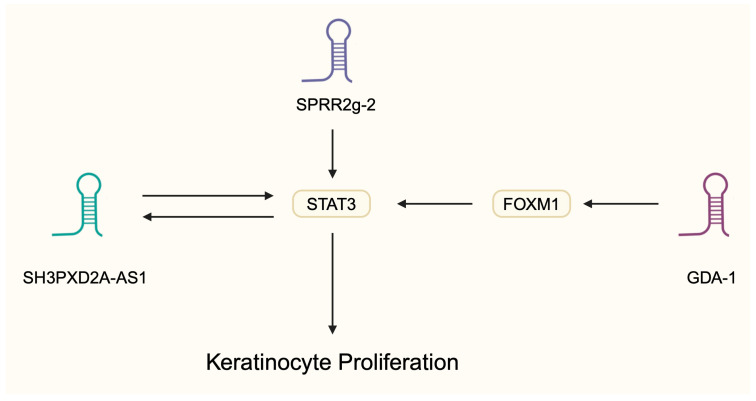
Long non-coding RNAs regulate the expression of STAT3 transcription factor to mediate keratinocyte proliferation. Created in BioRender. Kiełbowski, K. https://BioRender.com/u84r183.

**Figure 3:**
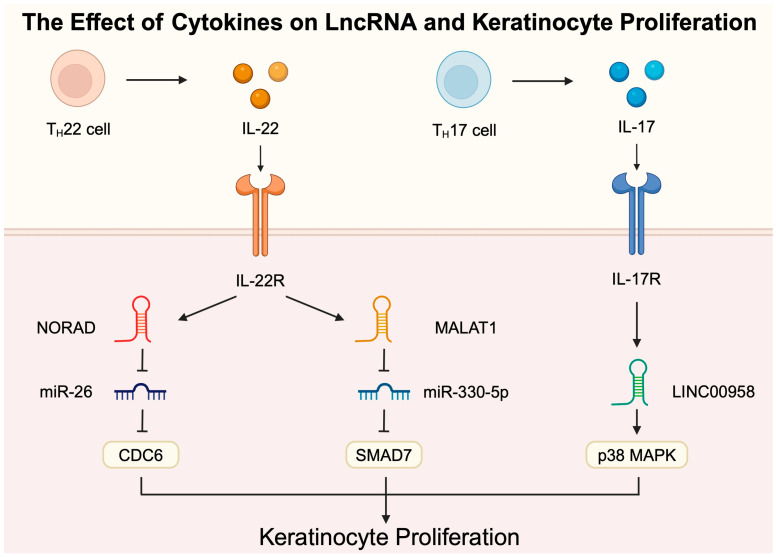
The involvement of long non-coding RNA in the IL-22- and IL-17-mediated stimulation of keratinocyte proliferation. Created in BioRender. Kiełbowski, K. https://BioRender.com/v29i018.

Some lncRNAs are implicated in interesting signaling loops. SH3PXD2A-AS1 is significantly upregulated in psoriasis skin tissue, and it was found to stimulate keratinocyte proliferation. Mechanistically, the lncRNA was suggested to enhance the expression of STAT3. By contrast, miR-125b could suppress its expression and it was negatively correlated with SH3PXD2A-AS1 expression. Interestingly, STAT3 can upregulate SH3PXD2A-AS1 [54], which demonstrates a signaling loop driving the progression of psoriatic lesions. These interactions are especially important considering STAT3’s activity in psoriasis pathogenesis. STAT3 is thought to be involved in the regulation of cytokines such as IL-21 and IL-17. It is important in Th17 cell differentiation and the expression of suggested autoantigen in psoriasis [55]. lnc-GDA-1 is another molecule that signals through STAT3 to promote keratinocyte proliferation. Specifically, Li and colleagues [56] recently demonstrated that lnc-GDA-1 is overexpressed in psoriatic tissue, and that it stimulates the expression of FOXM1, which activates STAT3. FOXM1 is a transcription regulator that plays an important role in cellular physiology. The altered expression of FOXM1 was identified in several diseases [57]. lnc-SPRR2G-2 also stimulates STAT3 to promote cell proliferation (Figure 2). Its overexpression and stimulation with static (STAT3 inhibitor) reduced the expression of pro-inflammatory mediators [58], thus further supporting the importance of interplay between inflammatory responses and keratinocyte behavior. The stimulation of keratinocytes with IL-17 enhance the expression of lnc-AGXT2L1-2:2, which stimulates the proliferation of skin cells. Mechanistically, the molecule acted through upregulating estrogen-related receptors [59], and estrogen was recently suggested to be involved in the promotion of psoriatic lesions [60]. Additionally, Luo et al. [61] demonstrated that the stimulation of keratinocytes with IL-17 enhances the expression of the lncRNA LINC00958, which promotes proliferation by regulating the p38 MAPK signaling pathway (Figure 3).

The lncRNA UCA1 is upregulated in psoriasis, and its expression decreases due to treatment with biologics, which suggests the involvement of the molecule in the pathophysiology of the disease. UCA1 is positively correlated with pro-inflammatory mediators such as IL-6 and CXCL1. Furthermore, it enhances the STAT3 and NF-κB signaling pathways [62].

lncRNA molecules induce protective mechanisms regarding keratinocyte proliferation as well. For instance, the expression of maternally expressed gene3 (MEG3) lncRNA is downregulated in psoriatic skin samples. It reduces the proliferation of keratinocytes by binding to miR-21. This interaction increases the expression of caspase-8 [63]. In addition, it is also involved in regulating inflammatory processes. Its overexpression suppresses the activity of PI3K/AKT/mTOR in TNF-stimulated keratinocytes, which was associated with suppressed inflammation [64]. It also enhanced the process of intracellular degradation and quality control known as autophagy. Aberrant autophagy was described in the development of psoriasis, and its modulation was suggested to impact the disease progression [65]. Autophagy-related 16-like 1 (ATG16L1) is a constituent of a substantial protein complex that is indispensable for autophagy [66]. Research has indicated that polymorphisms in the ATG16L1 gene are associated with an elevated risk of developing plaque psoriasis, and that ATG16L1 expression is augmented in the dendritic cells of patients with psoriatic arthritis [67,68]. The ATG16L1 protein has been found to form a complex with the ATG5 and ATG12 proteins, playing a crucial role in the elongation and closure of autophagosomal membranes, which are structures surrounding damaged organelles and pathogens [69].

The lncRNA TRAF3IP2-AS1 influences the activity of IL-17. The molecule is an anti-sense lncRNA of the *TRAF3IP2* gene, which encodes the IL-17 adaptor, Act1. TRAF3IP2-AS1 downregulates the expression of Act1, thus suppressing IL-17 signaling. The introduction of the TRAF3IP2-AS1 homolog to the IMQ psoriasis mouse model reduced the severity of skin lesions and epidermis thickness. Importantly, the treatment reduced myeloid cell infiltration, together with the levels of pro-inflammatory cytokines [70], demonstrating the benefits of lncRNA-based therapy. Other lncRNAs also affect IL-17 expression and signaling, which was examined in other disease models. These molecules include lncRNA-MIAT [71] and CASC2 [72], among others. lncRNAs also mediate Th17 cell differentiation, thus indirectly affecting IL-17 secretion [73].

IL-36 is another cytokine regulated by lncRNAs. The cytokine is involved in the progression of psoriasis, as it upregulates other pro-inflammatory cytokines and stimulates the infiltration of immune cells towards the skin. Keratinocytes demonstrate high expression of IL-36 receptors. Binding of the cytokine with the receptor induces downstream MAPK and NF-κB signaling [15]. The LINC01176 molecule sponges miR-218-5p to influence the expression of IL-36G. The introduction of sh-LINC01176 in to a mouse model of psoriasis reduced disease lesions, together with the levels of pro-inflammatory cytokines and IL-36G [74]. Furthermore, the knockdown of the CALML3-AS1 and SNHG9 molecules reduced activation markers of dendritic cells, drivers of psoriasis progression [75].

The increased expression of nuclear paraspeckle assembly transcript 1 (NEAT1) in the skin cells of psoriasis patients affects the activity of transcription factors during epithelial differentiation [76]. Recently, Wang et al. [77] found that the elevation of NEAT1 expression is associated with the reduced migration and proliferation of psoriatic keratinocytes, thus demonstrating the importance of altered NEAT1 expression in the pathophysiology of the disease. Researchers also demonstrated that NEAT1 stimulated the expression of galectin-7 (Gal7) [77], a β-galactoside-binding protein. The expression of Gal7 is reduced in psoriasis, which has been linked to the increased secretion of pro-inflammatory mediators [78]. Table 1 summarizes the expression of selected lncRNAs in patients with psoriasis.

## 3. Benefits and Limitations

Studies performed over the years offer a novel view on the pathogenesis of psoriasis. lncRNAs play important roles in skin functionality, such as keratinocyte differentiation [88]. The altered expression of lncRNAs, which is frequently observed in psoriasis, significantly contributes to keratinocyte impairment and enhanced inflammatory reactions, thus driving the progression of the disease. Increasing knowledge about the pathophysiology of psoriasis should aim to improve therapeutic strategies. For instance, the identification of crucial cytokines and molecules involved in the progression of psoriasis eventually led to the development of IL-17 inhibitors [89], TYK2 inhibitors [90], and IL-36 inhibitors [91], among others. It is possible that understanding the complex regulatory network of lncRNAs in psoriasis will introduce novel therapies. However, the use of ncRNA-based therapeutics is associated with certain limitations. To date, agents targeting miRNA represent the most widely known ncRNA therapeutics. In an article by Winkle et al. [92], the authors elegantly summarized agents whose further development was stopped. The reasons included lack of clinical efficacy, high costs, or toxicities. Furthermore, the development and implementation of lncRNA-based therapeutics is difficult due to the potential context-dependent activity of these molecules. Some studies demonstrate conflicting results regarding the immunoregulatory functions of lncRNAs. The molecules exert various cellular functions, as they can bind RNA, DNA, and proteins [18], which broadens their potential activity. Additionally, the significant number of lncRNAs suggests the characterization of functional groups, which could be monitored together. Analyses of lncRNAs expression could be implemented for diagnostic purposes or to evaluate treatment response. Nevertheless, studies with large populations are urgently required for the monitoring of lncRNAs in clinical practice. Thus, studies on lncRNAs offer an exciting novel view on the pathogenesis of psoriasis, but more large studies are required to translate these findings into the clinic.

## 4. Conclusions

Psoriasis is a disease with a complex pathophysiology involving interactions between immune cells and keratinocytes. lncRNA molecules are frequently dysregulated in patients or animal models of psoriasis. Since the discovery of PRINS and its potential involvement in psoriasis, numerous other lncRNA molecules were identified and investigated. Importantly, psoriasis is associated with hundreds or even thousands of differently expressed lncRNAs and the individual role of the majority of these molecules remain unknown. However, extensive research performed in recent years has proven the importance of lncRNAs in the pathogenesis of psoriasis. Their dysregulated expression can mediate keratinocyte proliferation and immune cell behavior and interactions with skin cells, hallmarks of psoriasis. In this paper, we did not focus on psoriatic arthritis, an important subtype of psoriasis involving the joints. lncRNAs are also involved in the pathogenesis of this articular condition [93]. Understanding the signaling pathways regulated by lncRNAs could result in more personalized treatment strategies that will eventually improve patients’ outcomes. Furthermore, monitoring circulating lncRNA levels could be implemented for diagnosis purposes or treatment evaluation.

## Figures and Tables

**Table 1:** Altered expression of long non-coding RNAs in both blood and skin biopsies from patients with psoriasis.

Material	lncRNA	Expression/Levels	Reference
Blood, skin biopsy	PRINS	Blood: downregulated Skin: upregulated	[34]
Blood	ANRIL	Up	[79]
Blood, skin biopsy	HOTAIR	Up	[80]
Skin biopsy	LINC00941	Down	[81]
Skin biopsy	LINC00958	Up	[61]
Skin biopsy	MEG3	Down	[63]
Skin biopsy	KLHDC7B-DT	Up	[82]
Skin biopsy	MALAT-1	Up	[49]
Skin biopsy	MIR31HG	Up	[83]
Skin biopsy	H19	Down	[84]
Skin biopsy	GAS5	Up	[85]
Skin biopsy	UCA1	Down	[86]
Skin biopsy	RP6-65G23.1	Up	[87]

## References

[1] Dopytalska K., Ciechanowicz P., Wiszniewski K., Szymańska E., Walecka I. (2021). The Role of Epigenetic Factors in Psoriasis. Int. J. Mol. Sci..

[2] Bu J., Ding R., Zhou L., Chen X., Shen E. (2022). Epidemiology of Psoriasis and Comorbid Diseases: A Narrative Review. Front. Immunol..

[3] Parisi R., Iskandar I.Y.K., Kontopantelis E., Augustin M., Griffiths C.E.M., Ashcroft D.M., Atlas G.P. (2020). National, regional, and worldwide epidemiology of psoriasis: Systematic analysis and modelling study. BMJ.

[4] Feldman S.R., Rastogi S., Lin J. (2018). Effect of Prior Biologic Use on Cost-Effectiveness of Brodalumab vs. Ustekinumab for Treatment of Moderate-to-Severe Psoriasis in the United States. Dermatol. Ther..

[5] Ho D., Koo E., Mamalis A., Jagdeo J. (2017). A Systematic Review of Light Emitting Diode (LED) Phototherapy for Treatment of Psoriasis: An Emerging Therapeutic Modality. J. Drugs Dermatol..

[6] Lee H.J., Kim M. (2023). Challenges and Future Trends in the Treatment of Psoriasis. Int. J. Mol. Sci..

[7] Koks S., Keermann M., Reimann E., Prans E., Abram K., Silm H., Koks G., Kingo K. (2016). Psoriasis-Specific RNA Isoforms Identified by RNA-Seq Analysis of 173,446 Transcripts. Front. Med..

[8] Song J.K., Yin S.Y., Li W., Li X.D., Luo Y., Xing M., Li B., Kuai L. (2020). An update on the role of long non-coding RNAs in psoriasis. Chin. Med. J..

[9] Zhou Q., Yu Q., Gong Y., Liu Z., Xu H., Wang Y., Shi Y. (2019). Construction of a lncRNA-miRNA-mRNA network to determine the regulatory roles of lncRNAs in psoriasis. Exp. Ther. Med..

[10] Gisondi P., Fostini A.C., Fossà I., Girolomoni G., Targher G. (2018). Psoriasis and the metabolic syndrome. Clin. Dermatol..

[11] Billi A.C., Gudjonsson J.E., Voorhees J.J. (2019). Psoriasis: Past, Present, and Future. J. Investig. Dermatol..

[12] Ogawa E., Sato Y., Minagawa A., Okuyama R. (2018). Pathogenesis of psoriasis and development of treatment. J. Dermatol..

[13] Jiang M., Fang H., Shao S., Dang E., Zhang J., Qiao P., Yang A., Wang G. (2019). Keratinocyte exosomes activate neutrophils and enhance skin inflammation in psoriasis. FASEB J..

[14] Xu X., Prens E., Florencia E., Leenen P., Boon L., Asmawidjaja P., Mus A.M., Lubberts E. (2021). Interleukin-17A Drives IL-19 and IL-24 Expression in Skin Stromal Cells Regulating Keratinocyte Proliferation. Front. Immunol..

[15] Sachen K.L., Arnold Greving C.N., Towne J.E. (2022). Role of IL-36 cytokines in psoriasis and other inflammatory skin conditions. Cytokine.

[16] Amin M., Lee E.B., Tsai T.F., Wu J.J. (2020). Psoriasis and Co-morbidity. Acta Derm. Venereol..

[17] Li X., Miao X., Wang H., Wang Y., Li F., Yang Q., Cui R., Li B. (2016). Association of Serum Uric Acid Levels in Psoriasis: A Systematic Review and Meta-Analysis. Medicine.

[18] Mattick J.S., Amaral P.P., Carninci P., Carpenter S., Chang H.Y., Chen L.L., Chen R., Dean C., Dinger M.E., Fitzgerald K.A. (2023). Long non-coding RNAs: Definitions, functions, challenges and recommendations. Nat. Rev. Mol. Cell Biol..

[19] Guttman M., Rinn J.L. (2012). Modular regulatory principles of large non-coding RNAs. Nature.

[20] Nedoszytko B., Szczerkowska-Dobosz A., Stawczyk-Macieja M., Owczarczyk-Saczonek A., Reich A., Bartosinska J., Batycka-Baran A., Czajkowski R., Dobrucki I.T., Dobrucki L.W. (2020). Pathogenesis of psoriasis in the “omic” era. Part II. Genetic, genomic and epigenetic changes in psoriasis. Postepy Dermatol. Alergol..

[21] Zhou Y., Li X., Duan Y., Luo Y., Tang S., Wang J. (2022). LncRNA MALAT-1 regulates the growth of interleukin-22-stimulated keratinocytes via the miR-330-5p/S100A7 axis. Autoimmunity.

[22] Danis J., Göblös A., Bata-Csörgő Z., Kemény L., Széll M. (2017). PRINS Non-Coding RNA Regulates Nucleic Acid-Induced Innate Immune Responses of Human Keratinocytes. Front. Immunol..

[23] Wu G.C., Pan H.F., Leng R.X., Wang D.G., Li X.P., Li X.M., Ye D.Q. (2015). Emerging role of long noncoding RNAs in autoimmune diseases. Autoimmun. Rev..

[24] Tsoi L.C., Iyer M.K., Stuart P.E., Swindell W.R., Gudjonsson J.E., Tejasvi T., Sarkar M.K., Li B., Ding J., Voorhees J.J. (2015). Analysis of long non-coding RNAs highlights tissue-specific expression patterns and epigenetic profiles in normal and psoriatic skin. Genome Biol..

[25] Stacey V.M., Koks S. (2023). Genome-Wide Differential Transcription of Long Noncoding RNAs in Psoriatic Skin. Int. J. Mol. Sci..

[26] Boehncke W.H., Schon M.P. (2015). Psoriasis. Lancet.

[27] Luo Y., Hara T., Kawashima A., Ishido Y., Suzuki S., Ishii N., Kambara T., Suzuki K. (2020). Pathological role of excessive DNA as a trigger of keratinocyte proliferation in psoriasis. Clin. Exp. Immunol..

[28] Kong S.M., Sun X.Y., Cui W.Y., Cao Y.C. (2023). Chemerin Exacerbates Psoriasis by Stimulating Keratinocyte Proliferation and Cytokine Production. Curr. Med. Sci..

[29] Qiao P., Guo W., Ke Y., Fang H., Zhuang Y., Jiang M., Zhang J., Shen S., Qiao H., Dang E. (2019). Mechanical Stretch Exacerbates Psoriasis by Stimulating Keratinocyte Proliferation and Cytokine Production. J. Investig. Dermatol..

[30] Richardson K.C., Aubert A., Turner C.T., Nabai L., Hiroyasu S., Pawluk M.A., Cederberg R.A., Zhao H., Jung K., Burleigh A. (2024). Granzyme K mediates IL-23-dependent inflammation and keratinocyte proliferation in psoriasis. Front. Immunol..

[31] Wu S., Zhao M., Sun Y., Xie M., Le K., Xu M., Huang C. (2020). The potential of Diosgenin in treating psoriasis: Studies from HaCaT keratinocytes and imiquimod-induced murine model. Life Sci..

[32] Aramwit P., Fongsodsri K., Tuentam K., Reamtong O., Thiangtrongjit T., Kanjanapruthipong T., Yadavalli V.K., Ampawong S. (2023). Sericin coated thin polymeric films reduce keratinocyte proliferation via the mTOR pathway and epidermal inflammation through IL17 signaling in psoriasis rat model. Sci. Rep..

[33] Tian S., Wang C. (2021). An ensemble of the iCluster method to analyze longitudinal lncRNA expression data for psoriasis patients. Hum. Genomics.

[34] Abdallah H.Y., Tawfik N.Z., Soliman N.H., Eldeen L.A.T. (2022). The lncRNA PRINS-miRNA-mRNA Axis Gene Expression Profile as a Circulating Biomarker Panel in Psoriasis. Mol. Diagn. Ther..

[35] Szegedi K., Göblös A., Bacsa S., Antal M., Németh I.B., Bata-Csörgő Z., Kemény L., Dobozy A., Széll M. (2012). Expression and functional studies on the noncoding RNA, PRINS. Int. J. Mol. Sci..

[36] Sonkoly E., Bata-Csorgo Z., Pivarcsi A., Polyanka H., Kenderessy-Szabo A., Molnar G., Szentpali K., Bari L., Megyeri K., Mandi Y. (2005). Identification and characterization of a novel, psoriasis susceptibility-related noncoding RNA gene, PRINS. J. Biol. Chem..

[37] Szegedi K., Sonkoly E., Nagy N., Nemeth I.B., Bata-Csorgo Z., Kemeny L., Dobozy A., Szell M. (2010). The anti-apoptotic protein G1P3 is overexpressed in psoriasis and regulated by the non-coding RNA, PRINS. Exp. Dermatol..

[38] D’Agostino M., Beji S., Sileno S., Lulli D., Mercurio L., Madonna S., Cirielli C., Pallotta S., Albanesi C., Capogrossi M.C. (2022). Extracellular Nucleophosmin Is Increased in Psoriasis and Correlates with the Determinants of Cardiovascular Diseases. Front. Cardiovasc. Med..

[39] Sun D.S., Guan C.H., Wang W.N., Hu Z.T., Zhao Y.Q., Jiang X.M. (2021). LncRNA NORAD promotes proliferation, migration and angiogenesis of hepatocellular carcinoma cells through targeting miR-211-5p/FOXD1/VEGF-A axis. Microvasc. Res..

[40] Li Y., Lv Y., Wang J., Zhu X., Chen J., Zhang W., Wang C., Jiang L. (2022). LncRNA NORAD Mediates the Proliferation and Apoptosis of Diffuse Large-B-Cell Lymphoma via Regulation of miR-345-3p/TRAF6 Axis. Arch. Med. Res..

[41] Wang K., Chen Z., Qiao X., Zheng J. (2023). LncRNA NORAD regulates the mechanism of the miR-532-3p/Nectin-4 axis in pancreatic cancer cell proliferation and angiogenesis. Toxicol. Res..

[42] Han Y., Tian H., Gao X. (2020). NORAD regulates proliferation and apoptosis in cardiomyocytes under high-glucose treatment through miRNA-150-5p/ZEB1 axis. Eur. Rev. Med. Pharmacol. Sci..

[43] Li S., Zhu X., Zhang N., Cao R., Zhao L., Li X., Zhang J., Yu J. (2021). LncRNA NORAD engages in psoriasis by binding to miR-26a to regulate keratinocyte proliferation. Autoimmunity.

[44] Li J., Pang D., Zhou L., Ouyang H., Tian Y., Yu H. (2024). miR-26a-5p inhibits the proliferation of psoriasis-like keratinocytes in vitro and in vivo by dual interference with the CDC6/CCNE1 axis. Aging.

[45] Staniszewska M., Kielbowski K., Rusinska K., Bakinowska E., Gromowska E., Pawlik A. (2023). Targeting cyclin-dependent kinases in rheumatoid arthritis and psoriasis—A review of current evidence. Expert. Opin. Ther. Targets.

[46] Sun S., Zhang X., Xu M., Zhang F., Tian F., Cui J., Xia Y., Liang C., Zhou S., Wei H. (2019). Berberine downregulates CDC6 and inhibits proliferation via targeting JAK-STAT3 signaling in keratinocytes. Cell Death Dis..

[47] Karner J., Wawrzyniak M., Tankov S., Runnel T., Aints A., Kisand K., Altraja A., Kingo K., Akdis C.A., Akdis M. (2017). Increased microRNA-323-3p in IL-22/IL-17-producing T cells and asthma: A role in the regulation of the TGF-beta pathway and IL-22 production. Allergy.

[48] Kielbowski K., Ptaszynski K., Wojcik J., Wojtys M.E. (2023). The role of selected non-coding RNAs in the biology of non-small cell lung cancer. Adv. Med. Sci..

[49] Elamir A.M., Shaker O.G., El-Komy M.H., Mahmoud Sharabi M., Aboraia N.M. (2021). The role of LncRNA MALAT-1 and MiRNA-9 in Psoriasis. Biochem. Biophys. Rep..

[50] Arun G., Aggarwal D., Spector D.L. (2020). Long Non-Coding RNA: Functional Implications. Noncoding RNA.

[51] Ghafouri-Fard S., Gholipour M., Abak A., Hussen B.M., Kholghi Oskooei V., Taheri M., Rakhshan A. (2022). Association analysis of MALAT1 polymorphisms and risk of psoriasis among Iranian patients. Int. J. Immunogenet..

[52] Masoumi F., Ghorbani S., Talebi F., Branton W.G., Rajaei S., Power C., Noorbakhsh F. (2019). Malat1 long noncoding RNA regulates inflammation and leukocyte differentiation in experimental autoimmune encephalomyelitis. J. Neuroimmunol..

[53] Xue Y., Ke J., Zhou X., Chen Q., Chen M., Huang T., Lin F., Chen F. (2022). Knockdown of LncRNA MALAT1 Alleviates Coxsackievirus B3-Induced Acute Viral Myocarditis in Mice via Inhibiting Th17 Cells Differentiation. Inflammation.

[54] Yang Z., Chen Z., Wang C., Huang P., Luo M., Zhou R. (2021). STAT3/SH3PXD2A-AS1/miR-125b/STAT3 positive feedback loop affects psoriasis pathogenesis via regulating human keratinocyte proliferation. Cytokine.

[55] Calautti E., Avalle L., Poli V. (2018). Psoriasis: A STAT3-Centric View. Int. J. Mol. Sci..

[56] Li X., Chen F., Ju J., Yin X., Yang Z., Li Z., Sun Q. (2023). Long Non-Coding RNA-GDA-1 Promotes Keratinocyte Proliferation and Psoriasis Inflammation by Regulating the STAT3/NF-kappaB Signaling Pathway via Forkhead Box M1. Inflammation.

[57] Zhang Z., Li M., Sun T., Zhang Z., Liu C. (2023). FOXM1: Functional Roles of FOXM1 in Non-Malignant Diseases. Biomolecules.

[58] Zhen Y., Li X., Huang S., Wang R., Yang L., Huang Y., Yan J., Ju J., Wen H., Sun Q. (2024). LncRNA lnc-SPRR2G-2 contributes to keratinocyte hyperproliferation and inflammation in psoriasis by activating the STAT3 pathway and downregulating KHSRP. Mol. Cell Probes.

[59] Wang R., Lin L., Lu X., Du J., Xu J. (2022). LncRNA AGXT2L1-2:2 facilitates keratinocytes proliferation and inhibits apoptosis by interacting with estrogen-related receptor alpha in psoriasis. Mol. Cell Probes.

[60] Wu H., Zeng L., Ou J., Wang T., Chen Y., Nandakumar K.S. (2022). Estrogen Acts Through Estrogen Receptor-beta to Promote Mannan-Induced Psoriasis-Like Skin Inflammation. Front. Immunol..

[61] Luo L., Pasquali L., Srivastava A., Freisenhausen J.C., Pivarcsi A., Sonkoly E. (2023). The Long Noncoding RNA LINC00958 Is Induced in Psoriasis Epidermis and Modulates Epidermal Proliferation. J. Investig. Dermatol..

[62] Hu Y., Lei L., Jiang L., Zeng H., Zhang Y., Fu C., Guo H., Dong Y., Ouyang Y., Zhang X. (2023). LncRNA UCA1 promotes keratinocyte-driven inflammation via suppressing METTL14 and activating the HIF-1alpha/NF-kappaB axis in psoriasis. Cell Death Dis..

[63] Jia H.Y., Zhang K., Lu W.J., Xu G.W., Zhang J.F., Tang Z.L. (2019). LncRNA MEG3 influences the proliferation and apoptosis of psoriasis epidermal cells by targeting miR-21/caspase-8. BMC Mol. Cell Biol..

[64] Tang Z.L., Zhang K., Lv S.C., Xu G.W., Zhang J.F., Jia H.Y. (2021). LncRNA MEG3 suppresses PI3K/AKT/mTOR signalling pathway to enhance autophagy and inhibit inflammation in TNF-alpha-treated keratinocytes and psoriatic mice. Cytokine.

[65] Roy T., Banang-Mbeumi S., Boateng S.T., Ruiz E.M., Chamcheu R.N., Kang L., King J.A., Walker A.L., Nagalo B.M., Kousoulas K.G. (2022). Dual targeting of mTOR/IL-17A and autophagy by fisetin alleviates psoriasis-like skin inflammation. Front. Immunol..

[66] Mizushima N., Kuma A., Kobayashi Y., Yamamoto A., Matsubae M., Takao T., Natsume T., Ohsumi Y., Yoshimori T. (2003). Mouse Apg16L, a novel WD-repeat protein, targets to the autophagic isolation membrane with the Apg12-Apg5 conjugate. J. Cell Sci..

[67] Liang N., Zhang K. (2024). The link between autophagy and psoriasis. Acta Histochem..

[68] Douroudis K., Kingo K., Traks T., Reimann E., Raud K., Rätsep R., Mössner R., Silm H., Vasar E., Kõks S. (2012). Polymorphisms in the ATG16L1 gene are associated with psoriasis vulgaris. Acta Derm. Venereol..

[69] Hamaoui D., Subtil A. (2022). ATG16L1 functions in cell homeostasis beyond autophagy. FEBS J..

[70] He R., Wu S., Gao R., Chen J., Peng Q., Hu H., Zhu L., Du Y., Sun W., Ma X. (2021). Identification of a Long Noncoding RNA TRAF3IP2-AS1 as Key Regulator of IL-17 Signaling through the SRSF10-IRF1-Act1 Axis in Autoimmune Diseases. J. Immunol..

[71] Qi Y., Wu H., Mai C., Lin H., Shen J., Zhang X., Gao Y., Mao Y., Xie X. (2020). LncRNA-MIAT-Mediated miR-214-3p Silencing Is Responsible for IL-17 Production and Cardiac Fibrosis in Diabetic Cardiomyopathy. Front. Cell Dev. Biol..

[72] Huang T., Wang J., Zhou Y., Zhao Y., Hang D., Cao Y. (2019). LncRNA CASC2 is up-regulated in osteoarthritis and participates in the regulation of IL-17 expression and chondrocyte proliferation and apoptosis. Biosci. Rep..

[73] He H., Qiu X., Qi M., Bajinka O., Qin L., Tan Y. (2022). lncRNA STAT4-AS1 Inhibited TH17 Cell Differentiation by Targeting RORgammat Protein. J. Immunol. Res..

[74] Zhao Z., Cheng J., Sun W., Zhu J., Lu S., Feng Y., Song Z., Yang Y., Wu X. (2024). The LINC01176-miR-218-5p-IL-36G Network is Responsible for the Pathogenesis of Psoriasis by Promoting Inflammation. Clin. Cosmet. Investig. Dermatol..

[75] Gao Y., Na M., Yao X., Li C., Li L., Yang G., Li Y., Hu Y. (2023). Integrative single-cell transcriptomic investigation unveils long non-coding RNAs associated with localized cellular inflammation in psoriasis. Front. Immunol..

[76] Fierro C., Gatti V., La Banca V., De Domenico S., Scalera S., Corleone G., Fanciulli M., De Nicola F., Mauriello A., Montanaro M. (2023). The long non-coding RNA NEAT1 is a ΔNp63 target gene modulating epidermal differentiation. Nat. Commun..

[77] Wang D., Cheng S., Zou G., Ding X. (2022). Paeoniflorin inhibits proliferation and migration of psoriatic keratinocytes via the lncRNA NEAT1/miR-3194-5p/Galectin-7 axis. Anticancer. Drugs.

[78] Chen H.L., Lo C.H., Huang C.C., Lu M.P., Hu P.Y., Chen C.S., Chueh D.Y., Chen P., Lin T.N., Lo Y.H. (2021). Galectin-7 downregulation in lesional keratinocytes contributes to enhanced IL-17A signaling and skin pathology in psoriasis. J. Clin. Investig..

[79] Rakhshan A., Zarrinpour N., Moradi A., Ahadi M., Omrani M.D., Ghafouri-Fard S., Taheri M. (2020). Genetic variants within ANRIL (antisense non coding RNA in the INK4 locus) are associated with risk of psoriasis. Int. Immunopharmacol..

[80] Yao X., Hao S., Xue T., Zhou K., Zhang Y., Li H. (2021). Association of HOTAIR Polymorphisms with Susceptibility to Psoriasis in a Chinese Han Population. Biomed. Res. Int..

[81] Ziegler C., Graf J., Faderl S., Schedlbauer J., Strieder N., Förstl B., Spang R., Bruckmann A., Merkl R., Hombach S. (2019). The long non-coding RNA LINC00941 and SPRR5 are novel regulators of human epidermal homeostasis. EMBO Rep..

[82] Yin X., Yang Z., Zhu M., Chen C., Huang S., Li X., Zhong H., Wen H., Sun Q., Yu X. (2022). ILF2 Contributes to Hyperproliferation of Keratinocytes and Skin Inflammation in a KLHDC7B-DT-Dependent Manner in Psoriasis. Front. Genet..

[83] Gao J., Chen F., Hua M., Guo J., Nong Y., Tang Q., Zhong F., Qin L. (2018). Knockdown of lncRNA MIR31HG inhibits cell proliferation in human HaCaT keratinocytes. Biol. Res..

[84] He Y., Yin X., Yan J., Li X., Sun Q. (2021). The lncRNA H19/miR-766-3p/S1PR3 Axis Contributes to the Hyperproliferation of Keratinocytes and Skin Inflammation in Psoriasis via the AKT/mTOR Pathway. Mediators Inflamm..

[85] Ahmed Shehata W., Maraee A., Abd El Monem Ellaithy M., Tayel N., Abo-Ghazala A., Mohammed El-Hefnawy S. (2021). Circulating long noncoding RNA growth arrest-specific transcript 5 as a diagnostic marker and indicator of degree of severity in plaque psoriasis. Int. J. Dermatol..

[86] Ma X.L., Wen G.D., Yu C., Zhao Z., Gao N., Liu Z.Y. (2021). LncRNA UCA1 negatively regulates NF-kB activity in psoriatic keratinocytes through the miR125a-A20 axis. Kaohsiung J. Med. Sci..

[87] Duan Q., Wang G., Wang M., Chen C., Zhang M., Liu M., Shao Y., Zheng Y. (2020). LncRNA RP6-65G23.1 accelerates proliferation and inhibits apoptosis via p-ERK1/2/p-AKT signaling pathway on keratinocytes. J. Cell Biochem..

[88] Zhang L., Piipponen M., Liu Z., Li D., Bian X., Niu G., Geara J., Toma M.A., Sommar P., Xu Landen N. (2023). Human skin specific long noncoding RNA HOXC13-AS regulates epidermal differentiation by interfering with Golgi-ER retrograde transport. Cell Death Differ..

[89] Camina-Conforto G., Mateu-Arrom L., Lopez-Ferrer A., Puig L. (2023). Bimekizumab in the Treatment of Plaque Psoriasis: Focus on Patient Selection and Perspectives. Patient Prefer. Adherence.

[90] Hoy S.M. (2022). Deucravacitinib: First Approval. Drugs.

[91] Bernardo D., Thaci D., Torres T. (2024). Spesolimab for the Treatment of Generalized Pustular Psoriasis. Drugs.

[92] Winkle M., El-Daly S.M., Fabbri M., Calin G.A. (2021). Noncoding RNA therapeutics—Challenges and potential solutions. Nat. Rev. Drug Discov..

[93] Dolcino M., Pelosi A., Fiore P.F., Patuzzo G., Tinazzi E., Lunardi C., Puccetti A. (2018). Long Non-Coding RNAs Play a Role in the Pathogenesis of Psoriatic Arthritis by Regulating MicroRNAs and Genes Involved in Inflammation and Metabolic Syndrome. Front. Immunol..

